# Supplementary material for: A Critical View of the Application of the APEX Software (Aqueous Photochemistry of Environmentally-Occurring Xenobiotics) to Predict Photoreaction Kinetics in Surface Freshwaters
Source: Molecules. 2019 Dec 18;25(1):9. doi: 10.3390/molecules25010009 (PMC7017383; doi:10.3390/molecules25010009)
Supplement: Supplementary file 1 [file molecules-25-00009-s001.zip › 3.2.4_gcc-4.4.0.zip!/3.2.4_gcc-4.4.0/3.2.4_gcc-4.4.0/doc/octave/html/interpreter/Temporary-Files.html]

Temporary Files - Untitled


Next: EOF and Errors,
Previous: Binary I/O,
Up: C-Style I/O Functions


---

#### 14.2.17 Temporary Files

Sometimes one needs to write data to a file that is only temporary.
This is most commonly used when an external program launched from
within Octave needs to access data. When Octave exits all temporary
files will be deleted, so this step need not be executed manually.

— Built-in Function: [fid, name, msg] = **mkstemp** (template, delete)  
> Return the file ID corresponding to a new temporary file with a unique
> name created from template. The last six characters of template
> must be `XXXXXX` and these are replaced with a string that makes the
> filename unique. The file is then created with mode read/write and
> permissions that are system dependent (on GNU/Linux systems, the permissions
> will be 0600 for versions of glibc 2.0.7 and later). The file is opened
> with the `O_EXCL` flag.
>
> If the optional argument delete is supplied and is true,
> the file will be deleted automatically when Octave exits, or when
> the function `purge_tmp_files` is called.
>
> If successful, fid is a valid file ID, name is the name of
> the file, and msg is an empty string. Otherwise, fid
> is -1, name is empty, and msg contains a system-dependent
> error message.
>
>
>
> **See also:** tmpfile, tmpnam, P\_tmpdir.

— Built-in Function: [fid, msg] = **tmpfile** ()  
> Return the file ID corresponding to a new temporary file with a unique
> name. The file is opened in binary read/write (`"w+b"`) mode.
> The file will be deleted automatically when it is closed or when Octave
> exits.
>
> If successful, fid is a valid file ID and msg is an empty
> string. Otherwise, fid is -1 and msg contains a
> system-dependent error message.
>
>
>
> **See also:** tmpnam, mkstemp, P\_tmpdir.

— Built-in Function: **tmpnam** (dir, prefix)  
> Return a unique temporary file name as a string.
>
> If prefix is omitted, a value of `"oct-"` is used.
> If dir is also omitted, the default directory for temporary files
> is used. If dir is provided, it must exist, otherwise the default
> directory for temporary files is used. Since the named file is not
> opened, by `tmpnam`, it is possible (though relatively unlikely)
> that it will not be available by the time your program attempts to open it.
>
>
>
> **See also:** tmpfile, mkstemp, P\_tmpdir.
